# Regularized group regression methods for genomic prediction: Bridge, MCP, SCAD, group bridge, group lasso, sparse group lasso, group MCP and group SCAD

**DOI:** 10.1186/1753-6561-8-S5-S7

**Published:** 2014-10-07

**Authors:** Joseph O Ogutu, Hans-Peter Piepho

**Affiliations:** 1 Bioinformatics Unit, Institute of Crop Science, University of Hohenheim, Fruwirthstrasse 23, 70599 Stuttgart, Germany

## Abstract

**Background:**

Genomic prediction is now widely recognized as an efficient, cost-effective and theoretically well-founded method for estimating breeding values using molecular markers spread over the whole genome. The prediction problem entails estimating the effects of all genes or chromosomal segments simultaneously and aggregating them to yield the predicted total genomic breeding value. Many potential methods for genomic prediction exist but have widely different relative computational costs, complexity and ease of implementation, with significant repercussions for predictive accuracy. We empirically evaluate the predictive performance of several contending regularization methods, designed to accommodate grouping of markers, using three synthetic traits of known accuracy.

**Methods:**

Each of the competitor methods was used to estimate predictive accuracy for each of the three quantitative traits. The traits and an associated genome comprising five chromosomes with 10000 biallelic Single Nucleotide Polymorphic (SNP)-marker loci were simulated for the QTL-MAS 2012 workshop. The models were trained on 3000 phenotyped and genotyped individuals and used to predict genomic breeding values for 1020 unphenotyped individuals. Accuracy was expressed as the Pearson correlation between the simulated true and the estimated breeding values.

**Results:**

All the methods produced accurate estimates of genomic breeding values. Grouping of markers did not clearly improve accuracy contrary to expectation. Selecting the penalty parameter with replicated 10-fold cross validation often gave better accuracy than using information theoretic criteria.

**Conclusions:**

All the regularization methods considered produced satisfactory predictive accuracies for most practical purposes and thus deserve serious consideration in genomic prediction research and practice. Grouping markers did not enhance predictive accuracy for the synthetic data set considered. But other more sophisticated grouping schemes could potentially enhance accuracy. Using cross validation to select the penalty parameters for the methods often yielded more accurate estimates of predictive accuracy than using information theoretic criteria.

## Background

Genomic prediction[1]is a method for predicting genomic breeding values for non-phenotyped individuals using molecular marker information covering the whole genome (e.g., Single Nucleotide Polymorphism, SNP) and observed phenotypic data from training populations. In essence, it involves a multiple regression of phenotypic observations on markers (SNP). The number of markers  typically runs into thousands and often far exceeds the number of phenotypes , leading to the classic  problem. The enormous number of markers involved in genomic prediction makes regularization methods particularly attractive and convenient tools for addressing the twin problems of selection of important markers and multicollinearity in the high dimensional regressions. In particular, the high dimensional nature of high-throughput SNP-marker data sets has prompted increasing use of the power and versatility of regularization methods in genomic selection to simultaneously select important markers and account for multicollinearity. Regularized (penalized) regression methods commonly used in genomic prediction include ridge [2], lasso (least absolute shrinkage and selection operator) [3], elastic net [4]and bridge [5]regression and their extensions [6,7].

These methods are not explicitly designed to exploit information on potential grouping structure among markers, such as that arising from the association of markers with particular Quantitative Trait Loci (QTL) on a chromosome or haplotype blocks, to enhance the accuracy of genomic prediction. The nearby SNP markers in such groups are linked, yielding highly correlated predictors. If such group structure is present but is ignored by using models that select individual predictors only, then such models may be inefficient or even inappropriate, leading to low accuracy of genomic prediction. Here, we explore if the accuracy of genomic prediction can be enhanced by explicitly accounting for potential grouping of SNP markers and using regularization methods with grouped penalties specifically designed to enable group selection. The predictive performances of the grouped methods are compared among the methods themselves and with those for corresponding but ungrouped variant of each method.

## Methods

### Linear regression model

Consider the linear regression model

(1) $$y_{i}=\beta_{0}\text{ + }\sum_{j=1}^{p}\beta_{j}x_{ij}\text{ + }\epsilon_{i},i=1,2,\ldots ,n$$

where $y_{i}$ is the *i*th observation of the response variable, $x_{ij}$ is the *i*th observation on the *j*th covariate, $\beta_{j}$ are the regression coefficients and $\epsilon_{i}$ are i.i.d. random error terms with $var\left(\epsilon \right)=I\sigma_{e}^{2}$, where  is the vector of *n *errors $\epsilon_{i}$ and  I is an *n*-dimensional identity matrix. In what follows we assume, without loss of generality, that the response and the covariates in (1) are mean-centered and standardized so that $\beta_{0}=0$, $\overset{n}{\underset{i=1}{\sum }}y_{i}=0,$$\overset{n}{\underset{i=1}{\sum }}x_{ij}=0$ and $\overset{n}{\underset{i=1}{\sum }}x_{ij}^{2}=1$[8]. In genomic prediction we are interested in estimating the  p regression coefficients $\beta_{j}$ which may be very many and many $\beta_{j}$ may be zero.

### Regularization methods

All regularized regression methods estimate the vector of regression coefficients  β in (1) by minimizing an objective function *F *composed of the sum of a loss function (e.g. the squared error loss=Residual Sum of Squares (*RSS*)) and a penalty function:

(2) $$F_{\lambda },\gamma \left(\beta \right)=\begin{array}{c} argmin\\ \beta \end{array}\left\{\underset{Loss\;Function=RSS}{\underset{︸}{\sum_{i=1}^{n}{\left(y_{i}-\sum_{j=1}^{p}\beta_{j}x_{ij}\right)}^{2}}}+\underset{Penalty\;function}{\underset{︸}{\sum_{j=1}^{p}p_{\lambda ,\gamma }\left(\beta_{j}\right)}}\right\},$$

where ${p_{\lambda }}_{,\gamma \left(.\right)}$ is a function of the vector of coefficients $\beta ={\left(\beta_{1},\beta_{2},\ldots ,\beta_{p}\right)}^{T}$ and the tuning(penalty) parameter λ>0 controls the tradeoff between minimizing the loss and the penalty terms. γ>0 is a shrinkage parameter that determines the order of the penalty function. Minimizing (2) yields a spectrum of solutions depending on the value of .

The gradient (first derivative) of a penalty function determines how it affects the solution in (2). To see this for bridge regression, consider the first derivative or rate of penalization of penalties of the form ${p_{\lambda }}_{,\gamma \left(\beta \right)}=\lambda \beta^{\gamma }$ with respect to  β, where  β is a scalar. In ridge regression $\left(\gamma =2\right)$, the rate of penalization $p_{\lambda ,\gamma }^{\prime }\left(\beta \right)=2\lambda \beta$ increases with  β, implying little or no penalization is applied when  β is near 0 but strong penalization is applied when  β is large. In lasso regression (γ=1), the rate of penalization $P_{\lambda ,\gamma }^{\prime }\left(\beta \right)=\lambda$ is constant. In bridge regression (e.g.,γ=1/2), the rate of penalization $p_{\lambda ,\gamma }^{\prime }\left(\beta \right)=\lambda /2\sqrt{\beta }$ is very high for values of  β near zero but declines rapidly as  β becomes large.

We consider the eight different regularized regression methods in turn below.

### Bridge regression

Bridge regression minimizes the penalized least squares objective function [5,9]

(3) $$Bridge_{\lambda }\left(\beta \right)=\;\begin{array}{c} argmin\\ \beta \end{array}\left\{RSS\text{ + }\lambda \sum_{j=1}^{p}{\left|\beta_{j}\right|}^{\gamma }\right\},$$

where *p*, $\beta_{j}$ and λ>0 and γ>0 are defined as in (2).

The optimal combination of  and  can be selected adaptively from the data by grid search using cross-validation. The bridge estimator is the value of  that minimizes (1) for any given γ>0[5,9]. The bridge estimator can do automatic variable selection since some coefficients become exactly zero when  and  is sufficiently large. For , a finite number of covariates and under appropriate regularity conditions, the bridge estimator (*i*) is consistent and (*ii*) can distinguish between covariates whose coefficients are exactly zero and covariates with nonzero coefficients in sparse high-dimensional settings [10].

[8]extended the results of [10] to infinite dimensional parameter settings (i.e. p→∞ as n→∞) and showed that the bridge estimator (*iii*) is selection consistent for any γ>0 and (*iv*) has the oracle property when 0<γ<1.The oracle property means that:[11,12]; (a) the bridge estimator correctly selects the nonzero coefficients with probability converging to 1 (i.e. with near certainty) and that (b) the bridge estimators of the nonzero coefficients are asymptotically normal with the same means and covariances that they would have if the zero coefficients were known in advance. The bridge estimator subsumes three important special cases. When (*v*)  γ=0 the bridge estimator (2) simplifies to the ordinary least squares estimator (subset selection). (*vi*) When  γ=1 the bridge estimator (2) reduces to the lasso estimator, which was introduced as a variable selection and shrinkage method [3].

(4) $$Lasso\left(\beta \right)=\;\begin{array}{c} argmin\\ \beta \end{array}\left\{RSS\text{ + }\lambda \sum_{j=1}^{p}\left|\beta_{j}\right|\right\}\text{ 0}\text{.}$$

(*vii*) When γ=2 the bridge estimator (3) simplifies to the ridge estimator (5) [1,13-15]

(5) $$Ridge\left(\beta \right)=\;\begin{array}{c} argmin\\ \beta \end{array}\left\{RSS\text{ + }\lambda \sum_{j=1}^{p}\beta_{j}^{2}\right\}$$

(*viii*) Since some components of the bridge estimator can be exactly zero when 0<γ<1 and  is sufficiently large, the bridge estimator can simultaneously estimate parameters and select variables in one step. (*ix*) The bridge estimator can adaptively select the penalty order (γ) from the data and produce flexible solutions in a range of settings. (*x*) Bridge estimators have demonstrated robust performance in various settings relative to other penalized regression methods, including the popularly used ridge regression, lasso and the elastic net [8]. For example, the bridge estimator correctly identifies zero coefficients with higher probability than do the lasso and elastic net estimators based on simulation results [8].

### MCP

The minimax concave penalty (MCP) is defined on $\left[0,\;\infty \right)$[16] as

(6) $${p_{\lambda }}_{,\gamma }\left(\beta \right)=\left\{\begin{array}{ccc} \lambda \beta -\frac{\beta^{2}}{2\gamma }, & if & \beta \leq \gamma \lambda ,\\ \frac{1}{2}\gamma \lambda^{2}, & if & \beta >\gamma \lambda \end{array}\right\}$$

where λ≥0 and γ>0. The expression for$p_{\lambda ,\gamma }\left(.\right)$ shows that MCP initially applies the same rate of penalization as the lasso does but continuously reduces the rate of penalization until the rate becomes 0 when β>γλ.

The MCP [17] is motivated by and is very similar to the smoothly clipped absolute deviation (SCAD, [11]) penalty function. The gradient of the SCAD penalty is given by [11]

(7) $$p_{\lambda ,\gamma }^{\prime }\left(\beta \right)=I\left(\beta \leq \lambda \right)+\frac{{\left(\gamma \lambda -\beta \right)}_{+}}{\left(\gamma -1\right)\lambda }I\left(\beta >\lambda \right)\text{ for some }\gamma >2\text{ and }\beta >0$$

This gradient function corresponds to a quadratic spline function with knots at  and γλ. The penalty functions for both MCP and SCAD are concave or nonconvex. Both MCP and SCAD aim to eliminate the unimportant predictors from the model while leaving the important predictors unpenalized. This is equivalent to fitting an unpenalized model in which the truly nonzero predictors are known beforehand (i.e. the 'oracle property'). MCP and SCAD are thus asymptotically oracle-efficient [11,17]. Accordingly, as n→∞, they select the correct regression model with probability tending to one and the non-zero coefficient estimates are asymptotically normal and have the same covariance matrix as if they were known in advance [11,18,19]. MCP performs well when there are many rather sparse groups of predictors, i.e., when the underlying model exhibits less grouping of predictors. MCP suffers when the non-zero coefficients are clustered into tight groups because it tends to select too few groups and makes insufficient use of the grouping information. SCAD has weaker grouping behaviour than the MCP [21]

### Group bridge, group lasso, sparse group lasso and group MCP methods

All the four grouped methods select the important groups of covariates. Group bridge, sparse group lasso and group MCP perform bi-level selection because they also identify the important members of each group [20,21]. Bi-level selection is appropriate if predictors are not distinct but have common underlying grouping structure. Bi-level selection differs from simple group selection in that in bi-level selection, variable selection is carried out at the group level and at the level of the individual covariates, resulting in the selection of important groups as well as members of those groups. But in group selection, only relevant groups are selected so that the estimated coefficients within each group will be either all zero or all nonzero.

The group bridge, sparse group lasso and group MCP penalties combine two nested penalties to enable bi-level selection.

### Group bridge

The group bridge estimator is [22,23]

(8) $$\text{g}Bridge_{\lambda }\left(\beta \right)=\;\begin{array}{c} argmin\\ \beta \end{array}\left\{RSS\text{ + }\lambda \sum_{l=1}^{L}C_{l}{∥\beta_{A_{l}}∥}_{1}^{\gamma }\right\}$$

where $A_{1},\ldots ,A_{L}$ are subsets of the set $\left\{1,\ldots ,p\right\}$ indexed by l=1,…,L and represent known groupings of the covariates, $\beta_{A_{l}}={\left(\beta_{j},j\epsilon A_{l}\right)}^{T}$ are the regression coefficients in the *l*-th group.λ>0 is the penalty parameter and $c_{l}\geq 0$ are constants that adjust the different dimensions of $\beta_{A_{l}}$ and assign different weights to the different coefficients. A simple choice of $c_{l}$ is $c_{l}\propto {{\left|A_{l}\right|}^{1-}}^{\gamma }$ where $\left|A_{l}\right|$ is the cardinality of $A_{l}$ (the length or number of unique elements in the set $A_{l}$). The group bridge penalty combines two penalties, namely the bridge penalty for group selection and the lasso penalty for within-group selection. The bridge penalty is applied on the *L*_1_-norms of the grouped coefficients in (8). The objective criterion (8) reduces to the standard bridge criterion (3) when $\left|A_{l}\right|=1$ and 1≤l≤L.

### Group lasso

The group lasso selects groups of variables but does not select individual variables within groups. The group lasso estimator is [22]

(9) $$gLasso\left(\beta \right)=\begin{array}{c} argmin\\ \beta \end{array}\left\{RSS+\overset{L}{\underset{l=1}{\sum }}{∥\beta_{A_{l}}∥}_{K_{l,2}}\right\}$$

in which $A_{l}$ and $\beta_{A_{l}}$(l=1,…,L) are defined as in (8), $K_{l}$ is a positive definite matrix and ${∥\beta_{A_{l}}∥}_{K_{l,2}}={\left(\beta_{A_{l}}^{T}K_{l}\beta_{A_{l}}\right)}^{\frac{1}{2}}$. [24] suggest using $K_{l}=\left|A_{l}\right|I_{l}$, where $\left|A_{l}\right|$ is the cardinality of $A_{l}$ and $I_{l}$ is the $\left|A_{l}\right|\times \left|A_{l}\right|$ identity matrix.

The reason that gLasso selects groups but not individual variables is made clearer by re-expressing (9) as [22]

(10) $$S\left(\beta ,\omega \right)=RSS+\sum_{l}^{L}\omega_{l}^{-1}{∥\beta_{A_{l}}∥}_{K_{l,2}}^{2}+\nu \sum_{l}^{L}\omega_{l}$$

Then minimizing $S\left(\beta ,\omega \right)$ subject to ω≥0 for some suitably chosen constant $\tilde{\omega }\geq 0$ yields $gLasso\left(\beta \right)$ in model (10) for appropriately chosen .

The objective criterion (10) reveals that gLasso behaves very much like an "adaptively weighted ridge regression" in which (*i*) the sum of the squared coefficients in group *l *is penalized by $\omega_{l}$, and (*ii*) the sum of the $\omega_{l}$'s is further penalized by . If ${\beta_{A}}_{l}=0$ when model (10) is minimized then group *l *is dropped from the model. But if ${\beta_{A}}_{l}\neq 0$ then all the elements of ${\beta_{A}}_{l}$ are nonzero and all the variables in group *l *are retained in the model [22].

Equivalently, the group lasso penalty can also be written as [25]

(11) $$gLasso\left(\beta \right)=\begin{array}{c} argmin\\ \beta \end{array}\left\{RSS+\sum_{l=1}^{m}\sqrt{p_{l}}{∥\beta_{l}∥}_{2}\right\}$$

where the loss (*RSS*) is computed using only observations of covariates in the submatrix of the matrix of all covariates with columns corresponding to covariates in group *l*, $\beta_{l}$ is the coefficient vector of that group and $p_{l}$ is the cardinality or length of $\beta_{l}$. The $\sqrt{p_{l}}$ terms account for the varying group sizes and ${∥.∥}_{2}$ is the Euclidean norm (not squared).

The group lasso estimator is asymptotically consistent even when model complexity increases with increasing sample size [26]. If only one variable is contained in each group then the objective function (9) simplifies to that of the usual lasso solution. gLasso penalizes the grouped coefficients much like the lasso does because it uses the same tuning parameter for all groups and hence suffers from estimation inefficiency and variable selection inconsistency. The adaptive group lasso remedies these shortcomings by applying different tuning parameters and hence different amounts of shrinkage to the grouped coefficients [27] much as the adaptive lasso does to individual covariates [18]. But the adaptive group lasso does not accomplish bi-level selection[28]. The group lasso over-shrinks individual coefficients when groups are sparsely populated.

### Sparse group lasso

The sparse group lasso ([25,29,30] also performs group-wise and within-group variable selection. The sparse group lasso penalty blends the lasso and group lasso penalties ([25,31]:

(12) $$SgLasso\left(\beta \right)=\begin{array}{c} argmin\\ \beta \end{array}\left\{RSS+\left(1-\alpha \right)\lambda \sum_{l=1}^{L}\sqrt{p_{l}}{∥\beta_{l}∥}_{2}+\alpha \lambda \sum_{l=1}^{pl}\left|\beta \right|\right\}$$

where  βis the full parameter vector, $\alpha \epsilon \left[0,1\right]$. Setting α=0 produces the lasso fit whereas α=1 yields the group lasso solution.

### Group MCP

The group MCP estimate minimizes [20,21]

(13) $$MCP\left(\beta \right)=\begin{array}{c} argmin\\ \beta \end{array}\left\{RSS+\sum_{l=1}^{L}\rho_{\lambda ,b}\left(\sum_{j=1}^{pl}\rho_{\lambda ,a}\left(\left|\beta_{lj}\right|\right)\right)\right\}$$

where  ρ is the MCP penalty (6), the tuning parameter of the outer penalty, *b*, is chosen to be $p_{l}a\lambda /2$ to ensure that the group level penalty attains its maximum if and only if all of its components are at their maxima, $p_{l}$ is the size of group *l,l *= 1,...,*L *groups and λ≥0 .

The group MCP therefore also combines two penalties to achieve bi-level, i.e., group and within- group variable selection. All the methods with grouped penalties make inflexible grouping assumptions that can undermine their performance when groups are misspecified or sparsely represented [20]. SCAD displays less grouping than group MCP and is thus expected to be less suited to grouped variable selection problems.

### Data set

An outbred population was simulated for the 16^th ^QTLMAS Workshop 2012. The simulation involved generating a base population (G0) of 1020 unrelated individuals (20 males and 1000 females) with a genome comprising 5 chromosomes, each having 2000 equally distributed SNPs. Each of the subsequent four non-overlapping generations (G1-G4) consisted of 20 males and 1000 females and was generated from the previous one by randomly mating each male with 51 females. Three milk production quantitative traits all of which express only in females were simulated. The traits were correlated and generated to mimic two yields and the corresponding content. Thus, the phenotypes, given as individual yield deviations, are only for the 3,000 females from G1 to G3. Young individuals (G4: individuals 3081 to 4100) have no phenotypic records. The pedigree of 4100 individuals, including the individual identity, sire, dam, sex and generation were provided as were the SNP genotypes for the 4100 individuals and the location of SNPs on each chromosome. Two alleles were given for each SNP. The marker information was coded as 1 for alleles $A_{1}A_{1}$, -1 for $A_{2}A_{2}$ and 0 for $A_{1}A_{2}$, or $A_{2}A_{1}$ and stored in a matrix $X=\left\{x_{ik}\right\}$, where $x_{ik}$ is the marker covariate for the  ith genotype $\left(i=1,\ldots ,G\right)$ and the  kth marker $\left(k=1,2,\ldots ,p\right)$. Monomorphic markers (*n *= 31) were identified and deleted prior to analysis, resulting in 10000-31 = 9969 markers. Here, we address only the second aim of the challenge which is to predict genomic breeding values for the 1020 unphenotyped progenies using the available genomic information.

#### Grouping SNP markers for the grouped methods

To enable model fitting for the grouped methods we formed groups of the markers by assigning consecutive SNP markers systematically to groups of sizes 1, 10, 20,...,100 separately for each of the five chromosomes. This often resulted in the last group having fewer SNPs than the actual prescribed group size. The total number of all groups of sizes 1, 10, 20,...,100 were 9969, 978, 490,...,100.

### Model fitting and selection

All the models were fit in R. Group lasso, group bridge, group MCP, and group SCAD models were fitted by the R packages *grpreg*. For each model and group size combination, the optimal value of  was selected by computing solutions along a grid of 100  values spaced evenly on the log scale following the approach of [31]. The value of  was fixed at its recommended default value in *gpreg *to reduce computing time to manageable levels. The Akaike (AIC) and Schwarz Bayesian (BIC) Information Criteria were used to select the optimal value of the penalty parameter  along the regularization path from the set of the 100  values for each model and group size combination [20]. The models with the selected best values for  for each group size were used to predict genomic breeding values for the 1020 unphenotyped genotypes. Pearson correlation between the predicted and true genomic breeding values was used to assess predictive accuracy. MCP and SCAD were also fitted to the ungrouped data using the R package *ncvreg *and the optimal value of  similarly selected from 100 values using 10-fold cross-validation. The 10-fold cross-validation involved partitioning the 3000 observations into 10 equal parts and estimating the prediction error in each set by using the observations in the other 9 sets to fit each of the models and predict the tenth part. Lastly, ridge regression was fitted to the ungrouped data in the R package *glmnet *net using 10-fold cross-validation.

## Results

The predictive accuracies attained by all the methods were mostly high. Although it improved prediction accuracy, overall grouping was not associated with a consistent increase in predictive accuracy (Tables 1 to 3). Nevertheless, the method used to select the penalty parameter (λ) often had a discernible impact on the accuracies of the regularization methods. The group bridge, group lasso and group MCP tended to produce better prediction accuracies with tuning parameters selected by AIC than by BIC. The sparse group lasso produced somewhat more accurate estimates than all the other methods for all the three synthetic traits. The best estimates of predictive accuracy for traits 2 and 3 were often slightly higher than the corresponding estimates for trait 1 (Tables 1 to 3). Results based on an alternative grouping of markers using *K*-means clustering (*K *= 10, results not shown) largely reproduced those for the systematic grouping and hence are omitted for the sake of brevity.

**Table 1:** Pearson correlation between the true and predicted genomic breeding values for group bridge, MCP, lasso and SCAD for trait T1 based on systematic groups.

Group size	Penalty selected by AIC					Penalty selected by BIC			
	**Bridge**	**MCP**	**Lasso**	**SCAD**	**SGLasso**	**Bridge**	**MCP**	**Lasso**	**SCAD**

1	0.682	0.758	0.778	0.767	0.781	0.682	0.768	0.773	0.652

10	0.770	0.759	0.793	0.788	0.787	0.753	0.772	0.747	0.735

20	0.774	0.761	0.788	0.770	0.787	0.777	0.772	0.756	0.667

30	0.758	0.760	0.790	0.774	0.787	0.787	0.771	0.753	0.644

40	0.769	0.761	0.789	0.758	0.787	0.771	0.772	0.754	0.595

50	0.774	0.761	0.780	0.740	0.791	0.763	0.770	0.732	0.579

60	0.765	0.760	0.784	0.750	0.791	0.765	0.771	0.706	0.581

70	0.771	0.760	0.779	0.760	0.789	0.757	0.770	0.718	0.619

80	0.781	0.761	0.776	0.759	0.795	0.747	0.770	0.721	0.550

90	0.761	0.760	0.774	0.748	0.781	0.743	0.770	0.709	0.478

100	0.771	0.760	0.778	0.706	0.790	0.760	0.770	0.704	0.502

Although listed under AIC, SGLasso used only 10-fold cross validation. The Pearson correlation for ridge regression for comparison is 0.737.

**Table 2:** Pearson correlation between the true and predicted genomic breeding values for group bridge, MCP, lasso and SCAD for trait T2 based on systematic groups.

Group size	Penalty selected by AIC					Penalty selected by BIC			
	**Bridge**	**MCP**	**Lasso**	**SCAD**	**SGLasso**	**Bridge**	**MCP**	**Lasso**	**SCAD**

1	0.756	0.790	0.826	0.810	0.841	0.828	0.837	0.827	0.795

10	0.779	0.809	0.852	0.840	0.845	0.819	0.839	0.813	0.776

20	0.762	0.809	0.844	0.833	0.845	0.818	0.838	0.779	0.780

30	0.758	0.809	0.836	0.827	0.845	0.801	0.838	0.765	0.744

40	0.710	0.810	0.818	0.788	0.845	0.790	0.838	0.735	0.688

50	0.714	0.809	0.827	0.808	0.846	0.807	0.837	0.746	0.697

60	0.708	0.808	0.810	0.804	0.843	0.810	0.837	0.738	0.708

70	0.700	0.809	0.806	0.804	0.843	0.789	0.837	0.731	0.680

80	0.702	0.809	0.811	0.800	0.844	0.790	0.837	0.735	0.641

90	0.669	0.808	0.795	0.793	0.848	0.785	0.837	0.714	0.607

100	0.704	0.808	0.803	0.792	0.841	0.798	0.837	0.808	0.632

Although listed under AIC, SGLasso used only 10-fold cross validation. The Pearson correlation for ridge regression for comparison is 0.772.

**Table 3:** Pearson correlation between the true and predicted genomic breeding values for group bridge, MCP, lasso and SCAD for trait T3 based on systematic groups.

Group size	Penalty selected by AIC					Penalty selected by BIC			
	**Bridge**	**MCP**	**Lasso**	**SCAD**	**SGLasso**	**Bridge**	**MCP**	**Lasso**	**SCAD**

1	0.811	0.818	0.818	0.796	0.807	0.812	0.813	0.814	0.716

10	0.742	0.818	0.835	0.816	0.814	0.742	0.813	0.776	0.763

20	0.776	0.818	0.814	0.794	0.814	0.766	0.813	0.742	0.735

30	0.801	0.818	0.818	0.818	0.814	0.791	0.813	0.742	0.742

40	0.812	0.818	0.804	0.797	0.814	0.790	0.813	0.725	0.725

50	0.809	0.817	0.814	0.818	0.816	0.801	0.813	0.716	0.716

60	0.825	0.817	0.802	0.813	0.817	0.808	0.813	0.712	0.712

70	0.822	0.816	0.806	0.816	0.815	0.806	0.813	0.710	0.710

80	0.824	0.816	0.795	0.788	0.816	0.807	0.813	0.686	0.686

90	0.803	0.818	0.793	0.776	0.816	0.817	0.813	0.665	0.598

100	0.820	0.817	0.791	0.764	0.797	0.760	0.813	0.776	0.665

Although listed under AIC, SGLasso used only 10-fold cross validation. The Pearson correlation for ridge regression for comparison is 0.762.

## Discussion

All the regularization methods produced consistent and relatively high estimates of predictive accuracy for all the three synthetic traits. The accuracies of all the estimates are such that each could potentially provide a firm basis for making practical selection decisions. Predictive accuracy varied with the method used to select the tuning or penalty parameter. There was some evidence that the group bridge, lasso, MCP and SCAD methods tended to produce somewhat more accurate estimates of predictive accuracy when the tuning parameter was selected by AIC than by BIC. This reinforces the suggestion of [32] that AIC-type criteria are often more appropriate if a model is used for prediction whereas BIC-type criteria are better suited for uncovering the true underlying model. Even so, the estimated predictive accuracy was sometimes decidedly higher when the tuning parameter was selected by 10-fold cross validation than by either of the information theoretic criteria. [33] recommend running cross-validation multiple times to obtain reliable results when small signals are expected. Accordingly, we ran the 10-fold cross validation 100 times, once for each of the 100 values of the tuning parameter for the grouped bridge, lasso, MCP and SCAD methods. For the sparse group lasso we replicated the 10-fold cross validation 20 times, once for each value of the tuning parameter. The observed improvement in predictive accuracy in some cases when using cross validation to select the penalty parameter is thus consistent with most of the markers having small signals.

There was no compelling evidence that grouping SNP markers consistently improved predictive accuracy for these data. This could mean either that the simulated SNP markers were not strongly correlated or that they indeed were but the simple systematic or K-means clustering grouping methods failed to accurately capture the underlying grouping structure. If the lack of clear improvement in performance is due to failure to accurately account for the underlying grouping structure then, assuming an accurate map information is available for each chromosome, using spatial clustering methods such as *K*-spatial clustering that partitions the genomic or chromosomal region into disjoint and contiguous intervals, subject to the constraint that SNPs in each group are spatially adjacent, and tagging these intervals with cluster numbers (1, 2,..., *K*), could potentially improve performance. If adjacent SNP markers are not independent, contrary to the assumption made by most common clustering frameworks, then spatial clustering should be more informative and more powerful than simple clustering of markers. A standard clustering procedure like *K*-means should perform poorly if markers are correlated because it ignores the genomic layout of the data and considers only the similarity of the SNP markers per loci. The grouped methods will also perform sub-optimally if the underlying grouping structure is too complex to accurately capture with simple clustering algorithms, including spatial clustering of groups. Such complexity may originate, for example, from overlapping of groups caused by SNPs linked to multiple QTLs.

The grouped methods we consider are not well suited to handling overlapping groups by construction. Extensions of the grouped methods would thus be needed to efficiently accommodate complications associated with overlapping groups. Existing extensions of the grouped methods designed to solve this type of complication include the overlapping group lasso that allows overlaps between groups of covariates. Some covariates are allowed to occur in more than one group but each time a covariate occurs in one group it gets a new coefficient [34,35]. This makes it possible to select one variable without selecting all the groups containing it. A related extension is the hierarchical (overlapped) group lasso that incorporates both main effects and interactions that obey weak or strong hierarchy (nesting) patterns [36-38]. To check if allowing for overlap among groups indeed improved predictive accuracy, we fitted the hierarchical group lasso model in the *glinternet *package in R and used10-fold cross-validation to select the optimal  value from a set of 50 values [38]. The estimated predictive accuracies of 0.759, 0.815 and 0.791 for traits 1, 2 and 3, respectively, showed that using overlapping groups did not improve accuracy relative to using non overlapping groups. Other extensions of the grouped methods applicable in slightly different settings include the group lasso for logistic regression [39], generalized linear models [40] and nonparametric models [41].

Although the performance of the different methods did not differ dramatically for these data the methods often differed with respect to their relative computational efficiencies. Other studies that have compared the performance of the group lasso with other grouped methods, for example, have also found similar results and more. In particular, [24] evaluated the performance of the group lasso relative to group Lars and group non-negative garrote. They found that the group lasso was the slowest of the three group methods because its solution path is not piecewise linear and hence requires intensive computations in large scale problems. The group Lars had comparable performance to the group lasso but was faster because its solution path is piecewise linear [24]. The group non-negative garrote cannot be directly applied to problems in which the total number of covariates exceeds the sample size because it depends explicitly on the full least squares estimates [24].

## Conclusions

All the methods produced relatively high estimates of predictive accuracy and hence can be used in genomic prediction research and practice. Systematic grouping or conventional *K*-means clustering of markers did not lead to any noticeable improvement in predictive accuracy. The grouped methods may yield better predictions with more sophisticated clustering approaches such as *K*-means spatial clustering which therefore deserve consideration in future studies. Whenever possible, the selection of the penalty parameter for the regularization methods should be done using replicated cross-validation to enhance accuracy of estimates. Nevertheless, selecting the penalty parameter using information theoretic criteria such as AIC and BIC may occasionally yield better estimates than cross-validation.

## List of abbreviations used

SNP: Single Nucleotide Polymorphism; QTL:Quantitative Trait Loci; RSS: Residual Sum of Squares; LASSO: Least Absolute Shrinkage and Selection Operator; MCP: The Minimax Concave Penalty; SCAD: Smoothly clipped absolute deviation; AIC: Akaike Information Criterion, BIC: Schwarz Bayesian Information Criterion.

## Competing interests

The authors declare that they have no competing interests.

## Authors' contributions

JOO conceived the study, conducted the statistical analysis and drafted the

manuscript. HPP read and edited the manuscript and oversaw the project. All the authors read and approved the manuscript.

## Declarations

The German Federal Ministry of Education and Research (BMBF) funded this research and publication within the AgroClustEr "Synbreed - Synergistic plant and animal breeding" (Grant ID: 0315526).

This article has been published as part of *BMC Proceedings *Volume 8 Supplement 5, 2014: Proceedings of the 16th European Workshop on QTL Mapping and Marker Assisted Selection (QTL-MAS). The full contents of the supplement are available online at http://www.biomedcentral.com/bmcproc/supplements/8/S5
